# Complete mitochondrial DNA sequence of the Japanese endemic catfish *Silurus lithophilus* (Siluriformes: Siluridae)

**DOI:** 10.1080/23802359.2021.1920486

**Published:** 2021-08-04

**Authors:** Yuu Kishimoto, Hisashi Okuyama, Jun-ichi Takahashi

**Affiliations:** Faculty of Life Sciences, Kyoto Sangyo University, Kyoto, Japan

**Keywords:** Next-generation sequencing, catfish, Lake Biwa, *Silurus lithophilus*, mitochondrial DNA

## Abstract

The Japanese endemic catfish *Silurus lithophilus* is distributed only in Lake Biwa, Lake Yogo, and their river drainages. There are four species of the genus *Silurus* in Japan, of which *S. lithophilus* has a limited distribution. This catfish needs to be collected for DNA data owing to the lack of information related to its conservation. Here, the complete mitochondrial genome of the *S. lithophilus* from Lake Biwa in Japan was analyzed using next-generation sequencing. The mitochondrial genome of *S. lithophilus* was identified as a 16,524 bp circular molecule containing 13 protein-coding genes (PCGs), 22 tRNA genes, and 2 rRNA genes, along with one A + T-rich control region. The AT content was 55.89%. The heavy (H)-strand was predicted to have 12 PCGs and 15 tRNA, and 2 rRNA genes, whereas the light (L)-strand was predicted to contain one PCG and seven tRNA genes. The start codons ATG and GTG were found in 13 PCGs. The stop codons TAA, TAG, and AGA were observed in all PCGs, except *CytB*. All tRNA genes formed typical cloverleaf secondary structures. The molecular phylogenetic relationship, inferred using 13 PCGs (based on the maximum likelihood), was consistent with that reported in previous studies, which predicted a sister relationship between *S. lithophilus* and *S. asotus*. The results also clearly indicated that *S. lithophilus* is a valid species.

The Japanese endemic catfish *Silurus lithophilus* Tomoda, 1961 is distributed in Lake Biwa, Lake Yogo, and their river drainages (Tomoda 1962; Maehata 2001, 2002). This species has a limited distribution, and information on its conservation is limited, including population size, life history, phylogenetic position, and genetic diversity. Mitochondrial DNA sequences can estimate phylogenetic relationships and genetic diversity. Although information on complete mitochondrial DNA sequences is abundant in several fish (Iwasaki et al. 2013), it is lacking in this species. Here, we report the complete mitochondrial genome of the *S. lithophilus* from Lake Biwa in Japan.

DNA sample from the fin muscle cells of *S. lithophilus* found in Lake Biwa (35°24′N 136°08′E) was immediately extracted using the DNeasy mini kit (Qiagen, Hilden, Germany) . DNA extraction for mitochondrial DNA analysis was based on the method of Tabata et al. (2016). The specimen was stored in the Shiga Prefectural Lake Biwa Museum, Japan (Specimen number1210058082). The gDNA library used for sequencing was prepared using the KAPA Hyper Prep kit, and a MiSeq sequencer (Illumina, San Diego, CA) was used to sequence the whole genome with an Illumina reagent kit. The gDNA library was indexed and run simultaneously over 600 cycles yielding paired reads of 250 bp.

The resultant reads were assembled and annotated using the Geneious R9 (Biomatters, Auckland, New Zealand) (Kearse et al. 2012) and MITOS web server (Bernt et al. 2013), respectively. Thirteen protein-coding genes (PCGs) sequences were aligned using Genetyx version 15 (GENEYTX, Tokyo, Japan) . The phylogenetic analysis (maximum likelihood analysis) was based on the nucleotide sequences of 13 PCGs using MEGA X (Kumar et al. 2018 ). The general time-reversible model and gamma-distributed with invariant sites were selected from the find best DNA program in MEGA X.

We succeeded in sequencing the entire mitochondrial genome of *S. lithophilus* from Lake Biwa, Japan. This sequence was given the DDBJ accession number LC574782. The genome comprised a 16,524 bp long closed loop, including 13 PCGs, 22 tRNA genes, 2 rRNA genes, and 1 AT-rich control region, similar to the typical catfish mitochondrial genomes (Nakatani et al. 2011; Vittas et al. 2011; ; Wang, Xu, Cui, et al. 2015; Wang, Xu, Xu, et al. 2015). The heavy (H)-strand was predicted to have 12 PCGs, 15 tRNA, and 2 rRNA genes, whereas the light (L)-strand was predicted to contain one PCGs and seven tRNA genes. All PCGs had ATG as the start codon, except *COI*, which had GTG as the start codon. As a stop codon, seven, four, and one gene used TAA, TAG, and AGA. Incomplete stop codons were identified in *CytB*. All tRNA genes formed typical cloverleaf secondary structures.

Phylogenetic analysis was performed using the sequences of 13 mitochondrial PCGs and those of 12 closely related taxa (Figure 1). The phylogenetic analysis suggested that *S. lithophilus* from Lake Biwa is most closely related to Japanese *S. asotus* and that *S. lithophilus* and *S. asotus* are sister species. This result is consistent with a published phylogenetic analysis inferred from the partial mitochondrial DNA sequence of *S. lithophilus* and *S. asotus* (Tabata et al. 2016). The results also clearly indicated that *S. lithophilus* is confirmed as a valid species of the genus *Silurus*.

**Figure 1.**
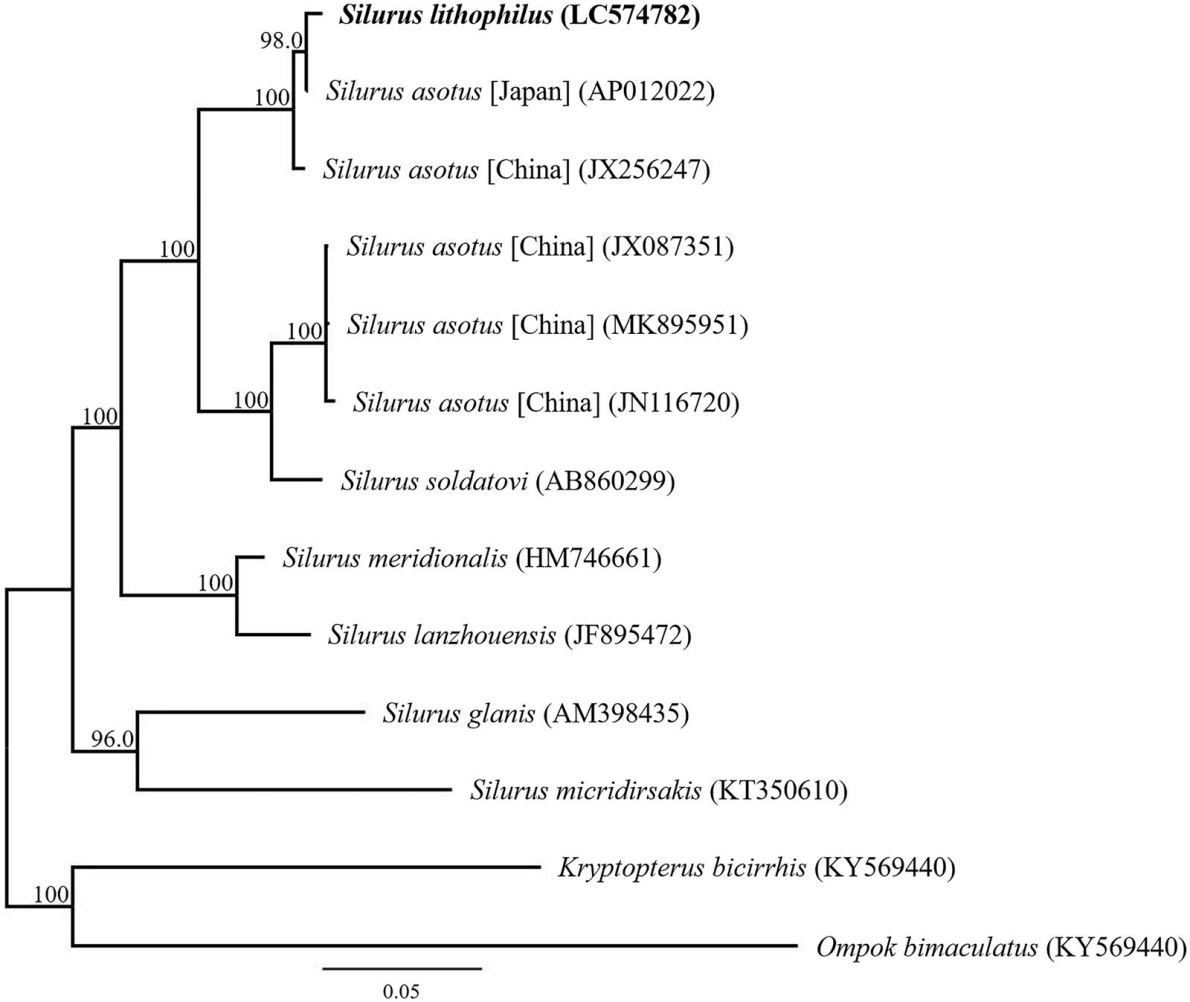
Phylogenetic relationships (maximum likelihood) of the catfish (Siluridae) based on the nucleotide sequences of the 13 protein-coding genes of the mitochondrial genome. The numbers at the nodes indicate the bootstrap support inferred from 1000 bootstrap replicates. Alphanumeric terms indicate the DNA Database of Japan accession numbers.

## Data Availability

The genome sequence data that support the findings of this study are openly available in DDBJ/GenBank at (https://www.ddbj.nig.ac.jp/index.html) under the accession no. LC574782. The associated BioProject ID, BioSample ID, and SRA (DRA) Accession no. are PRJDB11343, SAMD00283506, and DRA011643, respectively.
